# How Women Evaluate Birth Challenges: Analysis of Web-Based Birth Stories

**DOI:** 10.2196/12206

**Published:** 2018-12-18

**Authors:** Yasmine L Konheim-Kalkstein, Talya Miron-Shatz, Leah Jenny Israel

**Affiliations:** 1 Division of Social Sciences Mount Saint Mary College Newburgh, NY United States; 2 Center for Medical Decision Making Ono Academic College Kiryat Ono Israel; 3 Winton Centre for Risk and Evidence Communication Cambridge University Cambridge United Kingdom; 4 Interdisciplinary Center Psychology Department Interdisciplinary Center Herzliya Israel

**Keywords:** patient-centered care, decision making, parturition, women’s health

## Abstract

**Background:**

Birth stories provide an intimate glimpse into women’s birth experiences in their own words. Understanding the emotions elicited in women by certain types of behaviors during labor and delivery could help those in the health care community provide better emotional care for women in labor.

**Objective:**

The aim of this study was to understand which supportive reactions and behaviors contributed to negative or positive emotions among women with regard to their labor and delivery experience.

**Methods:**

We sampled 10 women’s stories from a popular blog that described births that strayed from the plan. Overall, 90 challenging events that occurred during labor and delivery were identified. Each challenge had an emotionally positive, negative, or neutral evaluation by the woman. We classified supportive and unsupportive behaviors in response to these challenges and examined their association with the woman’s emotional appraisal of the challenges.

**Results:**

Overall, 4 types of behaviors were identified: informational inclusion, decisional inclusion (mostly by health care providers), practical support, and emotional support (mostly by partners). Supportive reactions were not associated with emotional appraisal; however, unsupportive reactions were associated with women appraising the challenge negatively (Fisher exact test, *P*=.02).

**Conclusions:**

Although supportive behaviors did not elicit any particular emotion, unsupportive behaviors did cause women to view challenges negatively. It is worthwhile conducting a larger scale investigation to observe what happens when patients express their needs, particularly when challenges present themselves during labor, and health care professionals strive to cater to them.

## Introduction

### Background

The experience of birth is an event that women anticipate and prepare for months in advance. It holds a place of real significance in their lives and that of their baby. Women often approach the birth experience with expectations and trepidation. This is frequently expressed in their birth plans. The most common element of birth plans is pain management [1], but women also express requests regarding atmosphere and postpartum events [2]. Women’s plans and wishes for their birth are often communicated to the health care providers to increase coordination around labor and delivery preferences [3]. Birth plans have been shown to empower women, lessen anxiety [4], and lead to greater satisfaction with the birth experience [5], particularly when fulfilled [6]. Furthermore, birth satisfaction is predicted by patient expectations being met [7].

The birth experience, like many other medical experiences, does not always unfold according to expectations. Labor, for example, may involve adverse reactions to medications, risk to the mother’s health, or evidence that the baby might be in distress. Any of these events may precipitate the need for an induction, cesarean delivery, or instrumental delivery. These unexpected medical challenges and deviations from the plan can have emotional consequences. Generally, 20% to 33% of women report birth as traumatic [8]. If a birth does not go according to plan, some women experience negative emotions [2]. When coupled with poor support and negative perceptions of care, these women are more likely to experience a traumatic birth [9,10]. A negative emotional experience can have far-reaching consequences, such as hindered cognitive development in children of women who suffered from postnatal depression [11].

The way patients appraise their experiences can influence their health. For example, motivating a person to appraise a stressful situation as a *challenge*, as opposed to a *threat*, leads to better cardiac outcomes and enhanced performance on a stressful task [12]. Appraising a situation less negatively is found to be an effective way of down-regulating negative emotion [13]. Resilient individuals, those able to bounce back from stress, are more likely to use positive appraisals [14], indicating that it is not just the medical challenge per se that determines how the person emotionally evaluates the situation.

Researchers have suggested that satisfaction in the face of challenging medical situations can be increased by patient-centered care. For example, Ford and Ayers [15] analyzed birth stories and found that level of support during birth affects women’s mood, anxiety, and perceived control more than stressful interventions (eg, instrumental delivery). Another study showed that pain and medical interventions do not affect birth satisfaction as much as the relationship between the caregivers and the patient [16].

### Goals of the Study

In this study, we decided to focus on the interactions that occur in the face of challenges during labor and delivery, as these are more amenable to change through medical training and guidelines than individual patient variables such as patient resilience. We aim to classify these interactions and analyze the association between them and the emotional appraisal of the presented challenges. To do this, we used birth stories women posted on the Web and examined how the interactions present in those stories subsequently influenced the emotions the women experienced.

Sharing information about pregnancy and birth on social media is common practice. A study found that 44.4% of the women surveyed voluntarily posted pregnancy-related information on Facebook roughly once a month, with the goals of documenting the pregnancy (21.3%) and getting advice (28.9%) [17]. Women often also share their birth experiences in a narrative to support other pregnant women, to record their memory of this important life event, or to process the trauma [18,19].

In this study, narratives that women posted on the Web to share with others were specifically selected when the woman’s experience did not go according to her formal or informal birth plan. We treated each challenge presented in the women’s narratives as a unit of analysis and examined the surrounding supports (or explicit lack thereof) and the ensuing emotional appraisals of these challenges. We used both existing literature as well as the instances of support in the narratives to create a classification system of patient-centered supportive behaviors during labor and delivery. By analyzing women’s birth experiences in their own words, our study aimed to create a classification system for support during birth as well as reveal how interactions with women in labor at moments of challenge may influence appraisal of those challenges.

### Hypotheses

We hypothesized that we would find examples of emotional support as well as evidence of its importance. Research has shown that physicians who offer emotional reassurance are more effective and appreciated than those who do not [20,21]. Practitioner’s empathy can even reduce the duration and severity of the common cold as well as quantitatively increase the immune system response [22]. A qualitative study of patient experience in 14 specialties at Mayo Clinic reveals “empathetic,” “humane,” “personal,” and “respectful” to be among the 7 most important attributes of an ideal physician [23]. A lack of emotional support is also influential: poor emotional support is related to dissatisfaction with the birth experience. In-depth interviews of 10 women who had unexpected birth stories revealed that many of these women felt emotionally uncared for [24]. Furthermore, patient perception of nurses being uncaring is associated with the mother experiencing a birth as traumatic [25].

We also hypothesized that health care providers would provide patients with informational support. Birth experiences are appraised more positively if events are explained, misconceptions are corrected, and questions are answered [26]. Effective physician-patient communication, including information exchange, is related to improved memory, adherence, physiological outcomes, and patient satisfaction [27].

Finally, we hypothesized that health care providers would include women in decision making and management of their medical experience as an additional measure of support. A core value of patient-centered care is the principle of shared decision making: important medical decisions are made by health care providers together with patients, with patients’ values and preferences, scientific research, and the physician’s clinical expertise being taken into consideration [28]. Not only is patient inclusion considered more ethical, but patient perception of decisional inclusion is also significantly and positively related to patient satisfaction, trust, and understanding [29]. In the case of childbirth, a feeling of control over the birth experience has been directly related to satisfaction [30]. When patients are included in decision making, their sense of personal control and satisfaction with the birth experience increases [16]. Just as decisional inclusion has emotional benefits, exclusion from decision making is associated with emotional difficulties in the patient. A qualitative study by Goldbort [24] found that women’s difficulties in feeling in control during unexpected birth experiences often stemmed from a lack of decision-making power. Data from a recent survey of 3000 women showed that women who were consulted before having changes made to their birth plan, and consented to those changes, were more satisfied than those who did not give their consent [31].

Our study explored whether these supports were given not only by health care providers, but also by partners who accompanied the women to birth.

Finally, a research question was whether women would mention additional kinds of support as being given or explicitly missing. In addition, we questioned whether these supports would be associated with the emotional appraisal of the challenges.

## Methods

### Sample

The sample for this study consisted of 10 birth stories, each publicly posted by different women on the internet blog, This West Coast Mommy [32]. This blog is the number 7 Google search result for “Sharing Birth Stories” out of over 8,000,000 hits on Google. This particular blog has about 60 stories archived and receives about 40,000 visitors a month (O Lasting, unpublished data, January 2018). This website encourages women to submit their birth story to share with other women in Canada and the United States. The instructions on the blog read:

I’m interested in all your stories: natural or medicated birth, vaginal or C-section or VBAC, home or hospital, premature or full-term, orgasmic birth or birth trauma, adoption or surrogate or miscarriage. Did everything go as planned or were your expectations smashed to pieces? What would you change or do the same next time around? I reserve the right to edit for length (please aim for somewhere between 400-1000 words) or clarity, but you have the final say in how your story is published.

The editor clarified that she does not screen out any stories and "lightly edits at times for length or clarity" (O Lasting, unpublished data, October 2017).

We chose the 10 most recent stories that mentioned a diversion from the birth plan between the onset of labor and the baby’s first feeding. Stories had a range of 638 to 1799 words (mean 1224.60, SD 454.20).

### Coding

After independently reading a handful of stories, an initial coding scheme was agreed upon to capture events, supports, and appraisals. After independently coding each story, 2 of the authors met to agree on any discrepancies to reach 100% agreement and further refine the coding scheme if needed. A third author provided feedback on the coding scheme and acted as an additional reviewer when coding discrepancies or questions arose. To ensure the specificity of the coding scheme, percent agreement was calculated on one of the stories (approximately 10% of the events) and was reported to be 86%.

Coding applied to the birth experience, which extended from the onset of labor or induction to the baby’s first feeding. Thus, neither interactions with the health care provider before labor nor postpartum events in the hospital after the baby’s first latch or bottle were included.

Our focus was challenges experienced during birth. As such, we did not consider events that were described by the woman as neutral or positive; events were identified as challenges when these reflected negative or unclear emotional valence. An example of a challenge was:

I woke up after a few solid few hours of sleep and the pain was back, just as intense as before the epidural. I was 6 cm dilated, and the contractions were horrible...

On the other hand, this is an example of an event that was not coded as a challenge:

I was 4 cm dilated when I was first examined at the hospital.

We recorded if medical action was taken in response to the challenge (eg, analgesia administered or blood pressure measured). We then coded supportive or unsupportive reactions in response to each challenge. We further coded for the woman’s appraisal of her physical state (physical resolution) after any supportive (or unsupportive) reactions. If the woman attached a conclusion regarding her physical condition to the challenge, such as “I felt better,” this would be coded as positive physical appraisal. Alternatively, “my back hurt even more” would be coded as a negative physical appraisal. If there was no physical appraisal, the event was coded as missing or “neutral” physical appraisal.

Thereafter, we coded for emotional appraisals after any supportive or unsupportive reactions. An expression of emotion (eg, fear or anger) or a summary evaluation of the challenge that carried a clear affective valence was considered an emotional appraisal. For example, if the woman expressed that she “felt empowered,” this would be coded as positive emotional appraisal. Alternatively, “I was terrified” would be coded as a negative emotional appraisal. If there was no emotional appraisal, the event would be coded as missing emotional appraisal or “neutral.” Events could also be coded for mixed emotional appraisal:

The minute she finished that sentence I instantly burst into tears. I was happy. I was petrified. I was anxious. I think I felt every single emotion all at once, right at that moment.

A given event could have both physical and emotional appraisals. Consider, for example:

It hurt so bad, so I got an epidural. It helped the pain, but I felt so guilty about my decision.

Here, a woman describes a positive physical appraisal but a negative emotional appraisal (she felt guilty).

### Ethics Approval

This research project received ethical approval from the Ono Academic College. Note that we analyzed women’s birth stories that were freely available on the Web. No identifying information on the women was provided to us and none is included in the analysis.

## Results

### The Challenges

The stories were a sample of diverse birth experiences (see Table 1 for the types of experiences found in each story). Although the majority of the stories mentioned a spontaneous start of labor at home (contractions and/or water broke), 4 out of the 10 births ended in unplanned cesarean sections, and 6 of the 10 involved artificial induction of labor. Of the 7 women who planned to avoid an epidural, 6 ultimately received an epidural.

Overall, 90 events (challenges) were identified in the 10 stories. Each story had between 2 and 18 events (mean=9). The challenge of discomfort (pain, nausea, and exhaustion) occurred in every story and made up 38% (34/90) of all events. Medical issues related to the mother (eg, epidural side effects, artificial induction, failure to progress, and deviations in blood pressure) contributed to 29% (26/90) of all events and appeared in every story. Medical issues related to the baby (eg, fetal positioning or fetal distress) presented in 6 stories and contributed to 13% of all events. Managing fear and concern (6/90, 7%), problems with monitors (4/90, 4%), having to wait (4/90, 4%), and challenging interactions with health care providers (4/90, 4%) were each found in 3 of the stories (see Table 2).

### The Reactions

Some challenges were handled by responding medically (eg, distributing medication or using a vacuum). In 27% (24/90) of the events, there was medical action taken in response to the challenge. See Table 3 for which reactions were given by whom.

Following 53% (48/90) of the challenges, a supportive or unsupportive reaction took place. On the basis of our hypotheses, we identified 3 different types of reactions that could either be supportive or unsupportive: informational (woman is given information or if unsupportive, woman lacks information or comprehension), decisional inclusion (woman is included in decision making or if unsupportive, woman explicitly reports being left out of the decision, not having control, or being dismissed), and emotional (validation, affirmation, or empathy expressed or if unsupportive, a disregard or dismissal of feelings). The birth stories also revealed an additional type of support: practical support*.* We coded practical support as assisting the mother in nonmedical ways. Examples of practical support could be giving water to the mother or getting the mother a pillow. Similar to the other categories, the mother could also have an unmet need for practical support.

Table 4 provides examples of the different reactions to challenges. Informational support was health care providers’ most common reaction to challenges. In the following example, the doctor provides informational support, explaining why an induction is needed. The woman is scared, but her partner steps in with emotional support, and she is able to accept the situation:

He told me that I needed to be induced as soon as possible because my high blood pressure was very dangerous for both me and my baby...I was so scared. I looked at Bryan, and he grabbed my hand when he saw the look on my face. He told me, “You are going to do so good.” That’s all I needed to hear. I agreed to the induction.

Story #1

The above example illustrates how different constituents tend to support the mother in different ways. In our analysis, emotional support was solely provided by partners, and 83% (10/15) of the instances of practical support were also provided by partners. In contrast, 95% (19/20) of instances of informational support and 73% (11/15) of instances of decisional inclusion were given by health care providers.

**Table 1:** Details of the ten birth stories.

Story		1	2	3	4	5	6	7	8	9	10
Words (mean 1224, SD 454.20), n		1799	1432	1622	688	640	638	1550	961	1642	1274
Events (mean 9, SD 3.94), n		18	9	9	10	9	7	7	2	10	9
**Intentions for birth**											
	Planned for natural birth (n=4^a^)	No	No	No	Yes	No	Yes	Yes	No	Yes	Yes
	Planned to avoid epidural (n=6)	Yes	No	No	Yes	No	Yes	Yes	No	Yes	Yes
**Actual birth**											
	Received epidural (n=8)	Yes	Yes	Yes	Yes	Yes	Yes	No	No	Yes	Yes
	Spontaneous start of labor (or water broke) at home (n=7)	No	Yes	Yes	Yes	Yes	Yes	Yes	No	No	Yes
	Induction (n=6)	Yes	Yes	No	Yes	Yes	No	No	No	Yes	Yes
	Emergency cesarean section (n=4)	No	No	No	No	Yes	No	No	Yes	Yes	Yes
Hemolysis, elevated liver enzyme, low platelet syndrome (n=3)		No	No	No	No	Yes	No	No	Yes	Yes	No
Paralysis (n=1)		No	No	Yes	No	No	No	No	No	No	No
Premature baby (n=2)		No	No	No	No	No	No	No	Yes	Yes	No

^a^n values refer to the number of stories that reported these events.

**Table 2:** Emotional and physical appraisals for various types of challenges.

Challenges		Patient appraisal				Examples of challenges
		No appraisal (neutral)	Negative	Positive	Mixed	
**Discomfort (N=34), n (%)**						
	Emotional resolution	29 (85)	3 (9)	2 (6)	0 (0)	“The magnesium drip was awful. I felt rotten. I was instantly weak, queasy, and hot. Hot as hell.”
	Physical resolution	26 (77)	3 (9)	5 (15)	0 (0)	
**Medical issues of mother (N=26), n (%)**						
	Emotional resolution	16 (82)	5 (19)	4 (15)	1 (4)	“The epidural failed and a spinal had to be done.”
	Physical resolution	19 (73)	3 (12)	4 (15)	0 (0)	
**Medical issues with the baby (N=12), n (%)**						
	Emotional resolution	7 (58)	2 (17)	2 (17)	1 (8)	“The nurse told us the baby was facing the wrong direction.”
	Physical resolution	8 (67)	2 (17)	1 (8)	1 (8)	
**Fear or overwhelmed (N=6), n (%)**						
	Emotional resolution	1 (17)	1 (17)	2 (33)	1 (17)	“I was unable to see my son...I was terrified wondering if he was doing okay and I was so afraid...”
	Physical resolution	4 (67)	0 (0)	1 (17)	0 (0)	
**Difficult interactions (N=4), n (%)**						
	Emotional resolution	3 (75)	1 (25)	0 (0)	0 (0)	“A nurse began to check my belly for sensation with an ice cube. ‘Can you feel this?’ ‘Yes.’ ‘No you can’t.’ ‘Wait, what?’ Why bother asking me if you aren’t going to believe my answers?”
	Physical resolution	2 (50)	1 (25)	1 (25)	0 (0)	
**Problems with monitor (N=4), n (%)**						
	Emotional resolution	3 (75)	0 (0)	1 (25)	0 (0)	“They were having trouble finding the baby’s heart rate...”
	Physical resolution	3 (75)	0 (0)	1 (25)	0 (0)	
**Waiting (N=4), n (%)**						
	Emotional resolution	2 (50)	2 (50)	0 (0)	0 (0)	“After waiting two hours in a small triage room, with not a single nurse checking in on me...”
	Physical resolution	2 (50)	1 (25)	1 (25)	0 (0)	

**Table 3:** Supportive and unsupportive interactions with different constituents.

Reactions		Doctor	Nurse	Midwife	Health care provider (unspecified)	Doula	Partner	Overall
**Supportive reaction, n (%)**								
	Decisional	2 (13)	1 (7)	1 (7)	7 (47)	2 (13)	2 (13)	15 (100)
	Informational	9 (45)	3 (15)	2 (10)	5 (25)	1 (5)	0 (0)	20 (100)
	Emotional	0 (0)	0 (0)	0 (0)	0 (0)	0 (0)	3 (100)	3 (100)
	Practical	0 (0)	0 (0)	0 (0)	1 (8)	1 (8)	10 (83)	12 (100)
**Unsupportive reaction, n (%)**								
	Decisional	2 (50)	1 (25)	0 (0)	1 (25)	0 (0)	0 (0)	4 (100)
	Informational	1 (17)	0 (0)	0 (0)	5 (83)	0 (0)	0 (0)	5 (100)
	Emotional	0 (0)	0 (0)	0 (0)	1 (100)	0 (0)	0 (0)	1 (100)
	Practical	0 (0)	1 (100)	0 (0)	0 (0)	0 (0)	0 (0)	1 (100)

**Table 4:** Examples of supportive and unsupportive reactions.

Type of supportive or unsupportive reaction	Supportive reactions^a^	Unsupportive reactions^a^
Informational	“He [the doctor] told me *that I needed to be induced as soon as possible because my high blood pressure was very dangerous for both me and my baby. He told me I needed a magnesium drip.”* [Story #1]	“While I was in Triage, the nurse discovered my blood pressure was extremely high. I was kept in Triage and not sent to a Labor and Delivery room. *To this day I do not really understand why,* but my best guess is they were afraid my baby would be in distress and I would have an emergency c-section.” [Story #7]
Decisional input	"The doctors came in to do an exam. *They said I was 4 cm and that they would like to start oxytocin, “but your birth plan...””My what? Pff, don’t liksten to that. Do what you think is right, just tell me about it first,” “Okay, we would recommend an epidural with the oxyto—” “Yep, let’s get that going.* [Story #2]	“I was told she couldn’t administer the epidural until I was not in a contraction. She began preparing, and *I said, “I’m contracting,” but she went ahead anyway*.” [Story #3]
Emotional	“I was so scared. I looked at Bryan, *and he grabbed my hand when he saw the look on my face. He told me, ‘You are going to do so good.’* That’s all I needed to hear. I agreed to the induction,” [Story #1]	“After waiting two hours in a small triage room, with *not a single nurse checking in on me* I was feeling such dread.” [Story #10]
Practical	“I woke up at one point and yelled, ‘I’m going to barf!’ *My husband came running over with a take-out french fry tray* just in time for the barf to hit it like a skateboard ramp and go spewing across everything.” [Story #2]	“...that situation they put me in was stressful: waiting with no updates, alarms ringing every 15 minutes, *strapped to a bed and needing to use the bathroom, a pillowcase full of towels as they were out of pillows*...” [Story #10]

^a^Quotes are taken directly from the birth story without editing. Italics denotes the reaction, or sometimes, as in the case of unsupportive reactions—lack thereof.

**Table 5:** Others’ reactions to challenges and patients' ensuing emotional appraisals.

Reactions		No appraisal (Neutral, N=61), n (%)	Positive appraisal (N=11), n (%)	Negative appraisal (N=15), n (%)	Mixed appraisal (N=3), n (%)
**Supportive reaction**		29 (48)	7 (64)	5 (33)	2 (66)
	Decisional	9 (15)	1 (9)	3 (20)	0 (0)
	Informational	11 (18)	4 (36)	2 (13)	1 (33)
	Emotional	0 (0)	2 (18)	0 (0)	1 (33)
	Practical	9 (15)	0 (0)	0 (0)	0 (0)
**Unsupportive reaction**		6 (10)	0 (0)	5 (3)	0 (0)
	Decisional	2 (3)	0 (0)	2 (13)	0 (0)
	Informational	3 (5)	0 (0)	3 (20)	0 (0)
	Emotional	1 (2)	0 (0)	0 (0)	0 (0)
	Practical	0 (0)	0 (0)	0 (0)	0 (0)
Medical action taken		21 (34)	2 (18)	1 (7)	0 (0)

The events were also coded for both emotional and physical appraisals following the challenge and reactions. Most of the events (61/90, 68%) concluded with no emotional appraisal (neutral). Some events (11/90, 12%) concluded with positive appraisal, some events (15/90, 17%) concluded with a negative emotional appraisal, and some (3/90, 3%) events concluded with clearly mixed emotions (mixed resolution).

Most (65/90, 72%) events did not have a physical appraisal, 5 events (6%) concluded with a positive physical appraisal (eg, pain got better and baby was delivered successfully), 10 events (11%) concluded with negative physical appraisal (eg, “stuck in bed” and overwhelming pain), and 1 event (1%) concluded with mixed physical appraisal. Table 5 shows how different appraisals were related to different types of support.

A given event could be coded as having any of the reactions above (or any combination thereof).

Whether or not a patient received positive support did not affect the type of appraisal attached to the event. However, if a patient received an unsupportive reaction, appraisal attached to the event was affected (Fisher exact test, *P*=.02; see Table 6).

**Table 6:** Proportions of different types of patient emotional appraisals resulting from different reactions.

Reaction		No appraisal (neutral)	Positive appraisal	Negative appraisal
**Supportive^a^** **reaction^b^, n (%)**				
	Reported	29 (71)	7 (17)	5 (12)
	Not reported	32 (73)	5 (11)	7 (16)
**Unsupportive reaction^c^, n (%)**				
	Reported	6 (55)	0 (0)	5 (46)
	Not reported	60 (74)	11 (14)	10 (12)

^a^Support includes informational, decisional, emotional, or practical.

^b^Fisher exact test, *P*=.66, *nonsignificant.*

^c^Fisher exact test, *P*=.02.

## Discussion

### Principal Findings

Our study examined 10 birth stories that included 90 total challenges to the women during the childbirth process. These challenges included facing dangerously high blood pressure, premature delivery, and pain too intense to continue with the medication-free birth plan. Using each challenge and the related reactions and emotional appraisals as the unit of analysis allowed us to closely examine the psychosocial antecedents of how these women in labor emotionally evaluated each challenge. By analyzing women’s birth experiences in their own words, our study offers a classification system for support during birth and highlights how avoiding unsupportive interactions with women in labor at moments of unexpected challenge is crucial in helping women give these challenges a neutral or positive emotional resolution.

Consistent with our hypotheses, as well as on emergent findings from the stories, we determined that there were 4 types of supportive (or unsupportive) interactions that could take place when a woman in labor is faced with a challenge: emotional, decisional, informational, and practical (the last category identified from the data). Each challenge is an opportunity to support the mother.

Providing support did not mean interfering with the medical course of events. It did, however, involve catering to the woman’s needs for information, decisional inclusion, practical assistance, or emotional reassurance. For example, when a woman who had an epidural needle inserted twice was informed that this occurred because she was shaking (information), she accepted the answer and the challenge was resolved with emotional neutrality. In our analysis, practical support was the most common support partners gave, whereas health care providers gave nearly all instances of informational support and most instances of decisional inclusion.

Labor and delivery are composed of a string of challenges that need to be managed dynamically and cannot always be anticipated ahead of time. Notably, the medical challenges women face do not always determine the emotional outcome. For example, challenges that involved medical threats to the baby were equally likely to be resolved with positive or negative appraisal. When challenges involved medical issues of the mother, emotional appraisals were also nearly balanced between positive and negative. Note that in both cases, emotional appraisals for most challenges were missing. This was interpreted as neutral resolution, suggesting that the woman may have accepted the challenge and moved on.

Our data suggest that when women are responded to in an unsupportive way, they are more likely to evaluate the challenge with negative emotion. When unsupportive reactions were reported, the emotional appraisal of the challenge was 3.7 times more likely to be negative than if no unsupportive reactions were reported. The result that unsupportive reactions carried more weight than supportive reactions on women’s emotional evaluations of the challenges is not surprising. Research has shown that humans have a tendency to react more strongly to and be more emotionally affected by negative events than positive events [33,34]. Our data show that the most common unsupportive reaction reported by women was a lack of informational support:

But...the baby wasn’t crying. They swept her up and rushed her down the hall to the nursery. I was so scared. I cried as I said over and over, ‘’Where’s my baby? Is she okay?” They kept telling me she just needed some extra help.

In this example, the woman is not given a proper answer and is distraught because of the lack of information.

Our results are somewhat aligned with research showing that negative interpersonal events, consisting mostly of lack of support (eg, being ignored and feeling unsupported or abandoned), were the strongest predictor of traumatic birth experiences [35]. A third of women report this [9]. Although challenging intrapartum events such as an unplanned cesarean can predict adverse responses to childbirth, this is not a necessary condition. Traumatic feelings can also arise following normal vaginal deliveries [36].

Thus, as our study suggests, **s**atisfaction is determined more by how the challenges that arise are handled than by the nature of the challenges themselves. Indeed, a meta-analysis of women’s experiences highlights that feelings of trauma can result from a lack of a relationship with health care providers, poor communication, and care that leads women to feel dismissed or out of control [37]. Although many women hear the phrase “all that matters is a healthy baby,” mounting evidence suggests that a patient-centered approach can improve medical outcomes [38] and increase satisfaction with the childbirth experience, ultimately leading to better mental health and family outcomes [39].

Given the unpredictability of birth, some health care providers are reluctant to *plan* the birth experience [5,40]. However, the research reviewed above suggests that an informed, empowered woman is beneficial to the childbirth process. Our study adds to this knowledge by showing that challenges that present during labor and delivery do not necessarily lead to a negative emotional appraisal. The provision of support serves to mitigate the inherent negative effect challenges can have.

### Limitations

Our study has a few limitations. First, the information that the women share in their birth stories is likely what was salient in their memory, and their interpretation of the events is not necessarily an accurate portrayal of the experience [41,42]. Nonetheless, previous work has indicated that a woman’s perception of what occurred is relatively accurate, even over time. For example, Simkin [43] found that women’s memories of labor, particularly the actions of doctors, nurses, and partners, are “generally accurate, and many are strikingly vivid.” Furthermore, research shows that patient perceptions of an event have importance. These perceptions linger, influencing emotions and potentially future decision making. For example, colonoscopy patients’ evaluation of the procedure was associated with their willingness to repeat the procedure in the future [44]. However, the data women share must be interpreted cautiously. It would be a mistake to only use women’s recollections to surmise what physicians are or are not doing.

Second, we only analyzed the birth experiences of 10 women, albeit in depth, and can hardly presume they are a representative sample of women giving birth. Our aim in this study was to look at challenges as subjectively defined by the women experiencing them. Our study provides careful insight into the ways in which support, even during a potentially unpleasant event such as having an epidural needle inserted twice, can be perceived as having an emotionally neutral resolution. Furthermore, there might be selection bias in that these women have chosen to share their birth stories on the Web. Firestone et al similarly found that a self-selection bias exists among internet-based birth cohort studies [45]. Another possible insertion of bias involves the types of women who post on internet blogs. A study by Chilukuri et al found that low-income pregnant women are less likely to access most internet technologies than women at high-income levels [46]. Although the hemolysis, elevated liver enzyme, low platelet (HELLP) syndrome occurred in 3 out of the 10 women in our sample, this does not reflect the general population, where HELLP syndrome occurs in about 0.7% of pregnancies [47].

Finally, we only coded from the onset of labor to the babies’ first feeding. We, therefore, did not code for support that happened after birth such as in a follow-up appointment. Simple validation of feelings about the loss of a plan might help women cope better, and that may have happened the day after delivery. It is known that debriefing after a traumatic birth experience may reduce negative appraisals [48,49]. Limitations notwithstanding, ours is an in-depth examination of the challenges women encounter during birth, as told of their own initiative and in their own words. This provides us a powerful way of glimpsing into the patients’ experience.

### Conclusions

Future research should examine how individual variables, such as need for control or need for cognition, might interact with support. Women are not all the same in how they respond to others’ attempts at supportive behavior. For example, 1 woman was overwhelmed by a tour of the neonatal intensive care unit the day she was going to give birth to a premature baby:

The very next day I was wheeled into the NICU [neonatal intensive care unit] unit for a tour. I do not think anything in this world could have prepared me for that. I was taken into Pod 6 where the NICU nurse wanted to show me the approximate size my son would be when he was delivered. The baby was so tiny and delicate and hooked up to so many machines! There were alarms going off everywhere with nurses assisting the babies in need. I held my breath to hold back the tears. When I was wheeled into my room I lost it. I was inconsolable.

For others, informational support was comforting. Our data showed that informational support was twice as likely to lead to positive appraisal than negative appraisal.

In conclusion, by analyzing women’s accounts of their birth experiences in their own words, our study offers a classification system for support during labor and delivery and highlights the need to avoid unsupportive interactions with women in labor when unexpected challenges present themselves. Although challenges are unavoidable, we hope to have made a small contribution toward making them less emotionally painful.

## Abbreviations

HELLP: hemolysis, elevated liver enzyme, low platelet
NICU: neonatal intensive care unit
